# Prevalence and Genetic Characteristics of Geographic Atrophy among Elderly Japanese with Age-Related Macular Degeneration

**DOI:** 10.1371/journal.pone.0149978

**Published:** 2016-02-26

**Authors:** Yoichi Sakurada, Seigo Yoneyama, Atsushi Sugiyama, Naohiko Tanabe, Wataru Kikushima, Fumihiko Mabuchi, Atsuki Kume, Takeo Kubota, Hiroyuki Iijima

**Affiliations:** 1 Department of Ophthalmology, Faculty of Medicine, University of Yamanashi, Chuo, Yamanashi, Japan; 2 Department of Epigenetics, Faculty of Medicine, University of Yamanashi, Chuo, Yamanashi, Japan; International University of Health and Welfare, JAPAN

## Abstract

**Objective:**

To investigate the prevalence and genetic characteristics of geographic atrophy (GA) among elderly Japanese with advanced age-related macular degeneration (AMD) in a clinic-based study.

**Methods:**

Two-hundred and ninety consecutive patients with advanced AMD were classified into typical neovascular AMD, polypoidal choroidal vasculopathy (PCV), retinal angiomatous proliferation (RAP) or geographic atrophy (GA). Genetic variants of *ARMS2* A69S (rs10490924) and *CFH* I62V (rs800292) were genotyped using TaqMan Genotyping Assays. The clinical and genetic characteristics were compared between patients with and without GA.

**Results:**

The number of patients diagnosed as having typical neovascular AMD, PCV, RAP and GA were 98 (33.8%), 151 (52.1%), 22 (7.5%) and 19 (6.6%), respectively. Of 19 patients with GA, 13 patients (68.4%) had unilateral GA with exudative AMD in the contralateral eye. Patients with GA were significantly older, with a higher prevalence of reticular pseudodrusen, bilateral involvement of advanced AMD and T-allele frequency of *ARMS2* A69S compared with those with typical AMD and PCV; although there were no differences in the genetic and clinical characteristics among patients with GA and RAP.

**Conclusions:**

The prevalence of GA was 6.6% among elderly Japanese with AMD. Patients with GA and RAP exhibited genetic and clinical similarities.

## Introduction

Age-related macular degeneration (AMD) is one of the leading causes of blindness among the elderly in the developed countries.[1,2] Advanced AMD has been divided into exudative or wet AMD, and geographic atrophy (GA) or dry AMD. Exudative AMD is characterized by the accumulation of intra- or subretinal fluid, hemorrhage and disciform scar due to choroidal neovascularization. GA is characterized by progressive loss of photoreceptors, retinal pigment epithelium (RPE) and choriocapillaris within the macula. GA usually arises in the parafoveal region and progresses slowly over the years to involve the foveal center [3,4], leading to severe central vision loss. Even when the foveal center is spared from atrophy, paracentral scotoma may significantly impair the quality of vision. Color fundus photography of eyes with GA demonstrates sharply delineated areas of depigmentation in the macula with visibility of underlying large choroidal vessels due to reduced melanin pigment in the RPE cells.[5] Although the pathogenesis of GA has not been fully elucidated, choroidal ischemia, oxidative stress and chronic inflammation have been considered to be involved in its development.[6,7] Among genetic factors involved in the etiology of AMD including GA, *complement factor H* (*CFH)* and *age-related maculopathy susceptibility 2*(*ARMS2*) are considered two major loci among the Japanese population.[8]*CFH* I62V (rs800292) has been shown to be a major risk for AMD in the Japanese, which is comparable to *CFH* Y402H (rs1061170) in the Caucasians.

While the prevalence of exudative AMD among different ethnicities was not found to be different in a recent meta-analysis, it has been reported that the prevalence of GA is significantly higher in Europeans (1.11%) compared with Africans (0.14%), Asians (0.21%), and Hispanics (0.16%).[9] Among the scant literature on the prevalence of GA in Asians, the Hisayama study in Japan demonstrated that the prevalence of GA was 0.2%.[10]

Reticular pseudodrusen (RPD) are a subtype of drusen that exhibits a yellowish interlacing pattern, which is different from soft drusen and is clearly visualized on red-free or blue-light fundus photography.[11] Several studies have demonstrated that RPD are associated with advanced AMD, and that retinal angiomatous proliferation (RAP) is most highly associated with RPD.[12–14]

In the present study, we report the prevalence and the genetic or clinical characteristics of GA among elderly Japanese with AMD in the clinic.

## Methods

We retrospectively studied the medical charts of 290 consecutive patients with newly diagnosed advanced AMD between April 2012 and March 2015 at the Macular Clinic, Department of Ophthalmology, University of Yamanashi Hospital. This study was reviewed and approved by the Ethics and Gene Analysis Committee in the Faculty of Medicine, University of Yamanashi (approval date: March 23, 2007; Research number 348), and adhered to the tenets of the Declaration of Helsinki. Written informed consent was obtained from all subjects.

All patients were of Japanese descent and had undergone comprehensive examination, including best-corrected visual acuity measurement, slit-lamp biomicroscopy with 78 D lens, color fundus photography, fundus autofluorescence (FAF), near-infrared reflectance(NIR), fluorescein angiography (FA), indocyanine-green angiography (ICGA) and spectral-domain optical coherence tomography (SD-OCT). FAF, NIR, FA, ICGA and SD-OCT images were obtained using a Spectralis® (HRA2 Spectralis, Heidelberg Engineering, Dossenheim, Germany).

Exudative AMD was classified into three subtypes, including typical neovascular AMD, polypoidal choroidal vasculopathy (PCV), and RAP, which were determined by the authors (Y.S. or H.I.) based primarily on the FA, ICGA and SD-OCT findings. Eyes with typical neovascular AMD exhibited classic- or occult-type CNV in FA without polypoidal lesions in ICGA and SD-OCT findings of CNV either in the subretinal space or beneath the RPE line. Eyes with PCV exhibited clusters of polypoidal dilation of the vessels with or without abnormal vascular networks in the superficial choroid in ICGA and irregularly elevated RPE line in SD-OCT images. Eyes with RAP exhibited retinal–retinal or retinochoroidal anastomosis in FA or ICGA and retinal swelling with or without RPE detachment in SD-OCT images.

**Fig 1:**
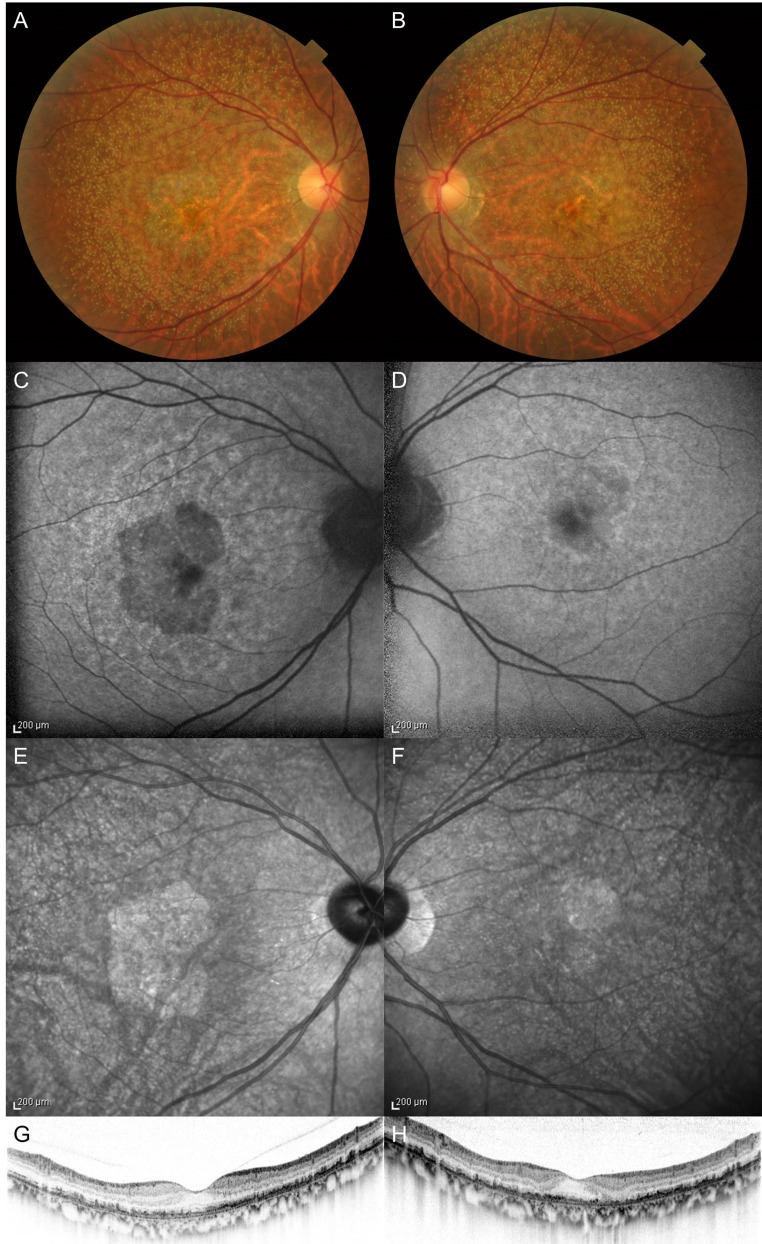
Multimodal imaging of geographic atrophy. (A) (B) Numerous reticular pseudodrusen and depigmentation of macular area were seen on color fundus photography. (C) (D) Fundus autofluorescence revealed hypoautofluorescence corresponding to geographic atrophy. (E) (F) Geographic atrophy was sharply delineated as hyperreflectance area by near infrared reflectance. (G) (H) Vertical line of spectral-domain optical coherence tomography demonstrated the loss of retinal pigment epithelium corresponding to geographic atrophy.

Eyes with GA were defined as exhibiting hypoautofluorescence with a minimum greatest linear dimension of at least 175 μm on FAF and disappearance of RPE on SD-OCT. Representative images of color fundus photography, FAF, NIR, SD-OCT associated with GA were shown in Fig 1.

The diagnosis of reticular pseudodrusen (RPD) was made by independent evaluators (Y.S. and S.Y.) based upon a specific reticular pattern around the macula, in which visibility was enhanced by IR, FAF, and upon SD-OCT findings of hyper-reflective subretinal deposits localized above the RPE layer. When the evaluators did not agree on the diagnosis of RPD, the final decision was made by a third evaluator (H.I.). Bilateral or unilateral involvement of advanced AMD was determined during the final visit to our clinic or on March 31 2015.

### Genotyping

Peripheral blood was collected from all patients. Genomic DNA was purified using a PUREGENE DNA Isolation Kit (Gentra Systems, Minneapolis, MN). Genotyping for *ARMS2* A69S (rs10490924) and *CFH* I62V (rs800292) was performed using TaqMan genotyping assays with 7300/7500 Real-Time PCR Systems (Applied Biosystems, Foster City, CA), according to the manufacturers’ recommendations.

### Statistical analysis

Statistical analysis was performed using DR SPSS for Windows (SPSS Inc., Tokyo, Japan). The overall allelic distribution was determined using 2×2 contingency tables. The differences of categorical variables between groups were evaluated by Fisher’s exact test. The differences of continuous variables between groups were evaluated by Mann-Whitney U test. P-values less than 0.05 were considered statistically significant.

## Results

The subjects were consecutive 290 patients with advanced AMD, comprised of 195 men (67.2%) and 95 women, aged 76.1±8.9 years. We divided the subjects into those with GA in at least one eye and those without GA in both eyes. The latter group was further classified into 3 subtypes of exudative AMD consisting of typical neovascular AMD, PCV and RAP. The demographic and genetic characteristics of the patients are shown in Table 1. Of 19 cases of GA, 5 (26.3%) were bilateral. The remaining 14 cases were unilateral for GA. However, 13 exhibited advanced exudative AMD in the associated eyes, including typical neovascular AMD (10 cases), PCV (one case) and RAP (2 cases).

**Table 1:** Demographic and genetic characteristic of patients with age-related macular degeneration according to subtypes.

	Typical neovascular AMD (n = 98)	PCV (n = 151)	RAP (n = 22)	Geographic atrophy (n = 19)
Mean age (years)	77.9±7.5	73.3±8.7	81.6±8.0	82.3±10.1
p-value (vs. GA)	0.026	1.2×10^−5^	0.72	NA
Gender Male	65(66.3%)	114(75.5%)	7(31.8%)	9(47.4%)
p-value (vs. GA)	0.12	9.8×10^−3^	0.31	NA
Presence of reticular pseudodrusen	19(19.4%)	2(1.3%)	12(54.6%)	14(73.7%)
p-value (vs. GA)	7.9×10^−6^	1.2×10^−14^	0.33	NA
Bilateral involvement of advanced AMD	19(19.4%)	12(8.0%)	18(81.8%)	18(94.7%)
p-value (vs. GA)	4.1×10^−10^	1.8×10^−15^	0.35	NA
*CFH* I62V(c.184G>A)(rs800292)				
GG	53(54.1%)	82(54.3%)	12(54.5%)	9(47.4%)
GA	40(40.8%)	56(37.1%)	10(45.5%)	9(47.4%)
AA	5(5.1%)	13(8.6%)	0	1(5.2%)
G-allele frequency	0.74	0.73	0.77	0.71
p-value (vs. GA)	0.69	0.85	0.62	NA
*ARMS2* A69S(c.205G>T)(rs10490924)				
TT	41(41.9%)	47(31.1%)	17(77.3%)	15(78.9%)
TG	45(45.9%)	73((48.4%)	4(18.2%)	3(15.8%)
GG	12(12.2%)	31(20.5%)	1(4.5%)	1(5.3%)
T-allele frequency	0.65	0.55	0.86	0.87
p-value(vs. GA)	7.2×10^−3^	1.7×10^−4^	1.0	NA

AMD: age-related macular degeneration, ARMS: age-related maculopathy susceptibility, CFH: complement factor H, PCV: polypoidal choroidal vasculopathy, RAP: retinal angiomatous proliferation

The clinical and genetic characteristics of the GA cases were compared with those of 3 subtypes of advanced exudative AMD shown in Table 1. The mean age of the GA patients was significantly higher than those with typical neovascular AMD or PCV, but was not significantly different from RAP (GA:82.3 ±10.1 years vs RAP:81.6±8.0 years, p = 0.72,Mann-Whitney U test). While the prevalence of reticular pseudodrusen of the GA patients was significantly greater compared with typical AMD or PCV, there was no significant difference in the prevalence of RPD between GA and RAP (GA: 73.7% vs RAP: 54.6%, p = 0.33, Fisher’s exact test).

While the *CFH* 162V-allele frequency of the GA cases was not significantly different from any of the subtypes of exudative AMD, the T-allele frequency of *ARMS2* A69S was significantly higher in GA patients compared with typical neovascular AMD or PCV though there was no significant difference in the T allele frequency of the *ARMS2* gene between GA and RAP (GA:87% vs RAP 86%,p = 1.0,. Fisher’s exact test)

## Discussion

While there have been a few reports investigating the prevalence of exudative AMD in the Asian population in clinical studies [15,16], there have been no reports regarding GA. To the best of our knowledge, this is the first report investigating the prevalence and clinical and genetic characteristics of GA among elderly patients with AMD in Asians. Maruko.et al reported that the prevalences of typical neovascular AMD, PCV and RAP were 35.3%, 54.7% and 4.5% respectively, in Japanese,[15] and the prevalence in the present study was similar: 33.8%, 52.1% and 7.5%, respectively.

RPD was more frequently observed in patients with GA (73.7%) compared with those with typical neovascular AMD (19.4%) or PCV (1.3%); however its prevalence was similar to those with RAP. Many investigators have reported that the subfoveal choroidal thickness is thinner in eyes with RPD than in those without.[17,18] Choroidal thinning could be considered an indication of choroidal ischemia, which might more greatly contribute to the development of RAP or GA than typical neovascular AMD or PCV. McBain et al.[19] reported that 19 eyes (36%) exhibited GA in 53 treatment-naïve eyes with RAP and that 57 eyes (86%) developed *de novo* GA or enlargement regardless of treatment modalities in 66 eyes with RAP. Their results may indicate that GA is a common feature in eyes with RAP and that RAP and GA may share a common pathophysiologic background.

In the present study, we genotyped two major loci of genetic markers associated with AMD in Japanese, *CFH* I62V (rs800292) and *ARMS2* A69S (rs10490924). The T-allele frequency of *ARMS2* A69S (rs10490924) was significantly different among eyes with exudative AMD with higher frequency in the order of RAP, typical neovascular AMD and PCV as reported previously. T-allele frequency in eyes with GA was similar to RAP and significantly higher compared with eyes with typical neovascular AMD or PCV. There have been two reports investigating distribution of the *ARMS2* variant in three subtypes. Tanaka et al. [20] have reported that T allele frequency of the *ARMS2* gene is 54.1%, 65.8% and 86.3% in PCV, typical neovascular AMD and RAP, respectively. Hayashi et al. [21] have reported that T allele frequency of the *ARMS2* gene is 54.8%, 64.4% and 90.3% in PCV, typical neovascular AMD and RAP, respectively. The current result was consistent with the previous reports.

A previous report conducting genome-wide association study for GA versus CNV in the Caucasians have revealed that the risk variant in *ARMS2* A69S is significantly higher in CNV group than in GA group[22], which is inconsistent with the present results. Though it was unclear why the results were different between studies, it might be involved in different ethnicities and selection of patients. In the previous report, patients having both GA and CNV were included in CNV group. Fuse et al. [23] genotyped A69S variant of *ARMS2* gene in 41 Japanese patients with GA and demonstrated that the risk allele frequency was 47.6%. This figure was much lower than the present results. To date, fundus autofluorescence (FAF) is an indispensable diagnostic tool for GA. The absence of FAF in the previous study might have lead to the results that are different from ours.

Previous studies of genotype-phenotype associations in eyes with AMD have reported that both bilateral involvement and coexisting RPD are closely associated with *ARMS2* variants. [13,14,24,25] In the present study, these two features were equally prevalent in patients with GA as in, those with RAP but were significantly higher than in those with typical neovascular AMD or PCV. Whether these results merely reflect the genetic factors or the presence of unknown pathologic factors should be investigated in future studies.

One major limitation of the present study is that it was conducted in a referral-based clinic, which might not precisely represent epidemiologic evidence. A second limitation is the small sample size of GA patients. Of 19 patients with GA, 13(68.4%) patients had GA and exudative AMD. This overlap might not reflect the features of GA, but complex advanced AMD. To confirm this speculatory conclusion, it will be necessary to conduct a large cohort study.

In conclusion, the prevalence of GA was 6.6% among elderly Japanese with AMD. Patients with GA exhibited clinical and genetic similarities with those with RAP.
